# Superior frontal and hippocampal structures associated with onset versus recurrence of mood disorders in monozygotic twins

**DOI:** 10.1038/s41380-026-03707-z

**Published:** 2026-06-23

**Authors:** Julian Macoveanu, Hartwig R. Siebner, Anjali Sankar, Lars Vedel Kessing, Maj Vinberg, Kamilla Woznica Miskowiak

**Affiliations:** 1 The Neurocognition and Emotion Across Disorders of the Brain (NEAD) Centre, Psychiatric Centre Copenhagen, Frederiksberg, Denmark; 2 Centre for Affective Disorders Research (CADIC), Psychiatric Centre Copenhagen, Frederiksberg, Denmark; 3 Danish Research Centre for Magnetic Resonance, Department of Radiology and Nuclear Medicine, Copenhagen University Hospital - Amager and Hvidovre, Copenhagen, Denmark; 4 Department of Clinical Medicine, Faculty of Medical and Health Sciences, University of Copenhagen, Copenhagen, Denmark; 5 Department of Neurology, Copenhagen University Hospital Bispebjerg and Frederiksberg, Copenhagen, Denmark; 6 Neurobiology Research Unit, Copenhagen University Hospital Rigshospitalet, Copenhagen, Denmark; 7 The Early Multimodular Prevention and Intervention Research Institution (EMPIRI), Mental Health Centre, Northern Zealand, Copenhagen University Hospital - Mental Health Services CPH, Copenhagen, Denmark

**Keywords:** Bipolar disorder, Depression, Predictive markers

## Abstract

Mood disorders involve complex neurobiological trajectories, making it difficult to identify predictors of their onset and progression. This prospective study disentangled markers of vulnerability from markers of illness recurrence by leveraging monozygotic twins discordant for mood disorders. We followed 136 monozygotic twins (66 affected [AT], 39 high-risk, and 31 low-risk unaffected twins [UT]; mean age 37.4 ± 9.1 years; 69.9% females) for 7.1 ± 0.8 years after baseline MRI. Cox regression examined associations between regional neuroanatomy (bilateral hippocampus, precuneus, anterior cingulate volumes; superior frontal gyrus [SFG] thickness) and: (1) time-to-first onset (UT) or (2) time-to-recurrence (AT), adjusting for age, sex, intracranial volume, and symptoms. In UT, thinner bilateral SFG and smaller left precuneus were associated with greater onset risk (HR = 1.1, p < 0.04). These regions showed an approximately linear relationship across structural quartiles, with progressively higher 7-year episode-free probability from Q1 (lowest measures) to Q4 (highest measures). In contrast, in AT, smaller precuneus and bilateral hippocampal volumes predicted higher recurrence risk (HR = 1.1, p < 0.037), following a threshold-like pattern, with markedly higher 7-year risk of a mood episode for individuals in Q1 (lowest volume) compared to the higher quartiles (Q2–Q4). Our findings indicate distinct neuroanatomical risk profiles: prefrontal thinning of SFG marks vulnerability to initial onset, potentially reflecting impaired cognitive control, while hippocampal atrophy predisposes to recurrence, possibly via stress-related neurotoxicity. These stage-specific biomarkers, identified under rigorous genetic control, highlight targeted prevention strategies: cognitive interventions preserving frontal integrity in high-risk populations, and hippocampal neuroprotection to sustain remission.

## Introduction

Mood disorders, notably major depressive disorder (MDD) and bipolar disorder (BD), are prevalent mental health conditions with significant global impact. These disorders exhibit complex phenotypic presentations, overlapping symptomatology, and shared neuroanatomical differences, complicating precise diagnosis and treatment [1]. Overlapping genetic liability, and the interplay of genetic and environmental risk factors contribute to the challenge of defining specific pathophysiological mechanisms underlying these disorders [2–4]. The genetic influence on susceptibility to mood disorders is well established, with studies estimating the heritability of MDD at up to 49% and BD at up to 85% [3, 5]. First-degree relatives of individuals diagnosed with mood disorders are two to ten times more likely to develop mood episodes than those with no family history [6, 7]. Notably, having a twin sibling with a mood disorder confers the highest risk, with concordance rates averaging 65% for monozygotic twins compared to only 14% for dizygotic twins [8]. Despite a growing body of neuroimaging studies investigating structural and functional markers of risk for onset or recurrence, findings remain inconclusive, partly due to a scarcity of longitudinal research.

Structural neuroimaging meta-analyses implicate alterations in large-scale neurocognitive networks in mood disorders: the central-executive network (CEN), default-mode network (DMN), and salience network (SN), which subserve cognitive control, self-referential processing, and affective valuation, respectively [9]. Structural deficits within these networks are most consistently reported in the hippocampus and precuneus (DMN), the dorsolateral prefrontal cortex (dlPFC; CEN), and the anterior cingulate cortex (ACC; SN) [10–16]. However, it remains unclear whether these cross-sectional differences reflect consequences of chronicity (e.g., medication effects, recurrent episodes) or whether they also index vulnerability prior to first onset. Prospective studies of high-risk populations, particularly monozygotic twins discordant for mood disorders, provide a powerful design to simultaneously control for shared genetic risk and distinguish neuroanatomical markers of initial onset (in unaffected co-twins) from those of recurrence (in affected twins), while minimizing confounding illness-related changes. For example, discordant twin designs from our group reveal that unaffected co-twins at familial risk exhibit smaller hippocampal volumes and greater prefrontal gray matter volumes (e.g., dorsal ACC, inferior frontal gyrus) compared to low-risk controls, a pattern that remained stable over a 7-year follow-up period [17, 18] and is consistent with observations in other young at-risk samples [19]. Studies of first-degree relatives extend these findings, reporting widespread cortical thinning or volume reductions in regions such as the dlPFC, ACC, insula, orbitofrontal cortex and the precuneus [12, 20, 21], along with smaller thalamic volume [22] and increased volume of the putamen [23], differences suggested to constitute vulnerability markers. Disturbances in amygdala development and in temporal, insular, and occipital cortices have also been associated with elevated risk for depression onset in adolescents [24]. Conversely, structural integrity of the amygdala [25] and aspects of the hippocampal morphology [26] have been proposed as markers of resilience. In our previous baseline study of the current cohort of monozygotic twins [26], the unaffected high-risk twins (UT) showed significantly larger bilateral hippocampal volumes, particularly in CA1-3, subiculum, and dentate gyrus subfields, compared to their affected twins (AT), suggesting that hippocampal enlargement may reflect a compensatory mechanism that buffers against onset. Nevertheless, the limited number of prospective longitudinal studies prevents clear determination of whether and to what extent these differences predict mood episode onset.

This study aimed to identify potential neuroanatomical predictors of mood disorder progression by examining two groups: monozygotic UT without baseline diagnoses (pooled from high-risk twins with affected co-twins and low-risk twins with no family history) and monozygotic AT in remission with prior mood disorder diagnoses. Using baseline structural MRI from our initial cross-sectional study [26] and 7-year follow-up clinical data, we employed Cox survival analyses to test whether baseline morphology of the hippocampus, precuneus, ACC, and the superior frontal gyrus (SFG), key hubs of the large-scale networks implicated in MDD and BD [10–16], predicted first-onset mood episodes in UT or recurrence in AT. To further contextualize these findings and enable a broader assessment of structural associations across the brain, we additionally conducted exploratory Cox survival analyses across the regions of the Desikan-Killiany cortical atlas [27], as well as vertex-wise cortical surface analyses comparing baseline cortical structure between twins who subsequently developed a mood episode and those who remained stable.

## Subjects and methods

The project was completed in accordance with the Declaration of Helsinki, and all participants gave written informed consent. The original baseline study [26] was approved by the regional ethical committee (H-3-2014-003), the Danish National Board of Health, and the Data Protection Agency (2014-331-0751). The current follow-up investigation was approved by the regional ethical committee (H-22022286).

### Sample and assessment at baseline

In total, 215 monozygotic twins between the ages of 18 and 50 were originally recruited between December 2014 and January 2017 after linking the Danish twin registry, the Danish Civil Registration System, and the Danish Psychiatric Central Register [28]. The record linkage identified monozygotic affected twins (AT) diagnosed with UD or BD according to ICD-10 and DF30-39 criteria between 1995 and 2014, their unaffected co-twins (high-risk UT), and unaffected twins with no family history of mood disorders (low-risk UT). Diagnoses were authenticated through the Schedules for Clinical Assessment in Neuropsychiatry (SCAN) interview [29]. The severity of the mood symptoms was evaluated using the Hamilton Depression Rating Scale 17 item version (HDRS-17) [30], the Young Mania Rating Scale (YMRS) [31], and the Major Depression Inventory (MDI) [32]. Remission or partial remission was indicated by scores <14 on either HDRS-17 or YMRS. The Edinburgh 10-item Inventory [33] was used to assess handedness. The clinical assessments, interview, and ratings were conducted by two researchers who were blind to the participants’ Danish Psychiatric Central Research Register diagnosis. Low-risk UT were excluded if they had any personal or first-degree relative with a history of organic mental disorder, schizophrenia spectrum disorder, or other mood disorders diagnosed from January 1995 to June 2014. The Family History Method diagnostic criteria was used when assessing history of psychiatric disorders in relatives. General exclusion criteria included prior head trauma with unconsciousness and subsequent effects, birthweight under 1300 g, pregnancy, ongoing substance abuse, severe somatic ailments, or dizygotic status. For more details on recruitment, screening, inclusion and exclusion criteria at baseline see [28].

### Assessment at follow-up

The follow-up assessment was conducted from July 2022 to April 2024, between 5.5 and 8.5 years following the baseline investigation. The twins were initially invited via their official personal digital mailbox (e-Boks), which is commonly used in Denmark for communication with public authorities. If there was no response within one week, the invitation was also sent to the personal email address provided during the baseline assessment. In the absence of a reply, participants received a follow-up email and were subsequently contacted by phone using the number provided at the baseline interview. Sixty-eight of the original participants declined to participate in the follow-up examination, two twins had died, and one who had emigrated. The twins who declined to participate were asked if they would be willing to complete a brief questionnaire. Clinical information for these individuals, including two who died and one that emigrated, was collected from The Danish Psychiatric Central Research Register, providing data on psychiatric hospital admissions. For more details see [34].

The twins who were included in the follow-up examination, underwent psychiatric evaluation, filled out questionnaires and were interviewed by a trained medical doctor. The clinical outcome was defined as onset of mood episodes for low-risk or high-risk unaffected twins, or recurrence during the follow-up period for twins affected at baseline. The onsets and recurrences were evaluated through a clinical interview and screened using the Mini-International Neuropsychiatric Interview (M.I.N.I.) [35] conducted by a trained MD. Validation and presence of psychiatric disorder for the participants with identified onset or recurrence of an affective episode during the follow-up period in the MINI interview was confirmed using the SCAN interview conducted by a blinded and SCAN-trained MD.

Of the participants included in the follow-up assessment, 136 had an MRI investigation at baseline and took part in the current analysis. This cohort included 66 affected twins (AT) in remission with a prior diagnosis of UD or BD at baseline, and 70 unaffected twins (UT) with no personal history of mood disorders at baseline. The UT sample consisted of a subsample of 39 twins with a co-twin diagnosed with UD or BD (high-risk) and a subsample of 31 twins who had no family history of mood disorders (low-risk).

### Cognitive assessment

Cognitive function was assessed at baseline and follow-up using the Screen for Cognitive Impairment in Psychiatry (SCIP) [36]. SCIP assesses cognitive function in five domains: Verbal Learning Test (VLT) – Measures immediate verbal memory, Working Memory Test (WMT) – Assesses working memory and attention, Verbal Fluency Test (VFT) – Evaluates verbal fluency and executive function, Delayed Verbal Recall (DVR) – Tests long-term memory retention, and Processing Speed (PS) – Measures cognitive processing speed.

### Statistical analyses of demographic, clinical, and cognitive data

Group means and standard deviations for demographic and clinical data at baseline were calculated for the UT (low- and high-risk combined) and AT groups. We assessed differences in these variables between clinical outcome subgroups defined according to presence or absence of onset or recurrence events within the UT and AT groups respectively using the Statistical Package for the Social Sciences (SPSS) version 29 (IBM, Armonk, New York, United States). Differences in discrete variables (sex, medication status, handedness, presence of other non-mood disorders) were assessed using Pearson’s chi-square tests. Continuous variables (age, education, illness duration/discordant time, time in remission, HDRS, YMRS) were assessed using mixed-model analysis of variance with group as fixed effect and twin pair index as random effect. For cognitive SCIP scores where both baseline and follow-up data were available, we applied a similar mixed models adding time as repeated factor and follow-up time as covariate and assessed group-by-time interaction effects. We report statistics of the group effect without correction for multiple comparisons to aid the interpretation of the imaging findings.

### Structural MRI processing and region of interest selection

MRI data were acquired using a 3T Siemens Verio scanner with a 32-channel head coil. The acquisition protocol included T1- and T2-weighted structural images; detailed MRI acquisition parameters are provided in the Supplemental Material. Cortical reconstruction and the subcortical volumetric segmentations were performed using the FreeSurfer image analysis suite v7.4.0 (http://surfer.nmr.mgh.harvard.edu/). For each subject, two T1- and one T2-weighted images corrected for B0 field geometric distortions were entered in the image analysis pipeline which included correction for intensity homogeneity and motion, intensity normalization, and automatic segmentation of the cortical and subcortical gray-matter structures as documented in [37, 38]. Surface-based data created by the processing pipeline included representations of cortical surface area, volume, and thickness. All FreeSurfer segmentations were visually inspected for accuracy. Manual edits were performed when necessary to correct pial surface misplacements and white matter topological defects, following standard FreeSurfer quality control procedures. All manual edits were carried out by a single trained research assistant who was blind to participant group membership and all other participant characteristics. Following each round of edits, FreeSurfer was rerun to integrate the corrections, and the output was reassessed to confirm adequate segmentation quality. Minor corrections were required and successfully applied in 93 of the 136 participants. We extracted the automatic volumetric segmentations including the total intracranial volume (TIV) and the cortical volume and thickness measurements from the parcellation performed using the Desikan-Killiany Atlas.

As primary predictors of interest, we selected the volumes of the bilateral hippocampus and precuneus, the ACC, and the thickness of the superior frontal gyrus targeting key limbic and cortical nodes of the DMN, SN and the CEN, respectively, based on their consistent implication across structural meta-analyses in mood disorders. Cox regression analyses were additionally conducted across all remaining regions of the Desikan-Killiany atlas and are reported as exploratory findings in the supplementary material.

### Cox survival analyses

Cox proportional hazards regression analyses were used to estimate hazard ratios (HR) for the proposed predictor variables’ influence on time to event (onset or recurrence). Separate survival analyses were run for each of the structural predictor variables in the UT and AT cohorts. All models were adjusted for age, TIV, and subclinical depression symptoms (HDRS-17) as continuous variables, and sex and risk status/diagnosis as categorical variables. Age, sex, and TIV were included to account for known influences on brain structure [39], whereas HDRS-17 scores were included to control for the potential effects of residual affective symptoms on risk estimates [40]. The models including UT were adjusted for risk status (low-risk/high-risk), and the models including the AT were adjusted for diagnosis (MDD/BD). The intra-twin pair dependence was corrected for by using a cluster effect (twin pair), accounting for within-pair correlation. Participants who emigrated or had change of diagnosis to DF00 - DF29 were right-censored at the date of the event. Participants who died during follow-up (*n* = 2) were included in the analysis and right-censored at their date of death (event = 0). Although censoring at death is technically informative, the small number of such cases makes it unlikely to meaningfully bias estimates. Assumptions of the Cox regression were evaluated by visual inspection of log-minus-log survival curves and by testing correlations between Schoenfeld residuals and survival time. No violations of the proportional hazards assumption were detected. Linearity of continuous covariates was assessed by inspecting plots of Martingale residuals against each continuous predictor, which showed no marked deviations from linearity. All continuous predictors and covariates were screened for outliers (z exceeding ± 3 SD). Two outliers were detected in the HDRS scores of the UT group; these cases were retained, as excluding them in a sensitivity analysis did not significantly affect the results. The SPSS statistical package was used to create a database and to undertake the statistical analyses. To improve interpretability, we expressed hazard ratios (HRs) for meaningful decreases in each brain measure. Using the model-estimated HR per original unit (1 mm³ for volumes, 1 µm for thickness), we translated the chosen decrease into the model’s measurement units and applied this transformation to the HR and 95% confidence intervals. Specifically, HRs are reported for a 100 mm³ decrease in subcortical volume and a 0.01 mm decrease in cortical thickness, calculated as:

$${{{\rm{HR}}}}_{{{\rm{unit}}}\; {{\rm{decrease}}}}={({{{\rm{HR}}}}_{{{\rm{per}}}\; {{\rm{original}}}\; {{\rm{unit}}}})}^{-\Delta },$$

where Δ is the size of the chosen unit. For comparability, HRs per 0.5 SD decrease (within UT and AT respectively) are reported in Supplementary Material. Analyses were performed separately for left and right hemispheres for each of the four regions of interest (i.e., eight ROIs in total) to enable detection of potential lateralization effects. The level of significance was set at p < 0.05 after correction for multiple comparisons. We applied Šidák correction using the effective number of independent tests (*k*_eff_) [41], an approach that estimates the true degree of independence among correlated tests and adjusts the correction accordingly, avoiding the overcorrection that would result from treating all ROI tests as fully independent. *K*_eff_ was calculated to 3.383 via the Variance Inflation Factor (VIF) approach, based on an average correlation of r = 0.195 among the tests (*n* = 8), yielding a corrected significance threshold of p < 0.015. As a sensitivity analysis we also report Benjamini–Hochberg false discovery rate (FDR) q-values in the Supplementary Material (Table S1). To further compare the relative contribution of different brain structures to the probability of remaining episode-free, we derived adjusted survival curves from the Cox proportional hazards model, including relevant covariates (age, sex, TIV, HDRS, and risk status/diagnosis). For visualization, structural predictors were grouped into quartiles, defined as low (Q1; ≤25th percentile), medium (Q2–Q3; 25th–75th percentile), and high (Q4; ≥75th percentile), and we plotted the predicted episode-free survival probabilities over time. The predicted 7-year survival probabilities for each quartile are also reported for all structures. Because quartile-based subgroups were defined separately for each brain structure, the corresponding sample sizes for each split are provided in the Supplementary Material. Unadjusted Kaplan–Meier survival curves for the original baseline groups of the twin cohort (low-risk UT, high-risk UT, and AT) were also generated, with statistical differences assessed using the log-rank test (p < 0.05).

### Post-hoc analyses

We examined whether the a priori selected brain ROIs tested in the Cox models predicted change in the cognitive domains that showed significant group (onset vs. no onset) × time interaction effects within the UT group. Linear mixed-effects models were fitted using baseline and follow-up cognitive scores as the outcome, with Time (baseline vs. follow-up), the baseline ROI volume, and their interaction entered as fixed effects. All models were adjusted for baseline age, sex, and TIV and accounted for non-independence within twin pairs.

For brain structures that were significant predictors in the main Cox regression analysis within the UT group, as a post-hoc sensitivity analysis, we assessed whether respective Cox analyses remained significant within the high-risk UT sample only.

### Exploratory cortical surface analysis

We performed vertex-wise surface-based analyses across the entire cortex to identify baseline structural differences between twins who subsequently developed mood episodes (onset in UT or recurrence in AT) versus those who remained stable. Using FreeSurfer, we constructed general linear models (GLMs) separately for each hemisphere to test group differences in cortical thickness and volume while controlling for age, sex, and total intracranial volume (TIV). Cortical surfaces were smoothed using a 5 mm full-width-at-half-maximum (FWHM) Gaussian kernel. Statistical significance was determined at p < 0.05 after cluster-wise correction for multiple comparisons via Monte Carlo simulation (using precomputed FreeSurfer data) with an absolute cluster-forming threshold of Z = 3.0 (p < 0.001).

## Results

### Demographic, clinical, and cognitive evaluation

**Fig. 1:**
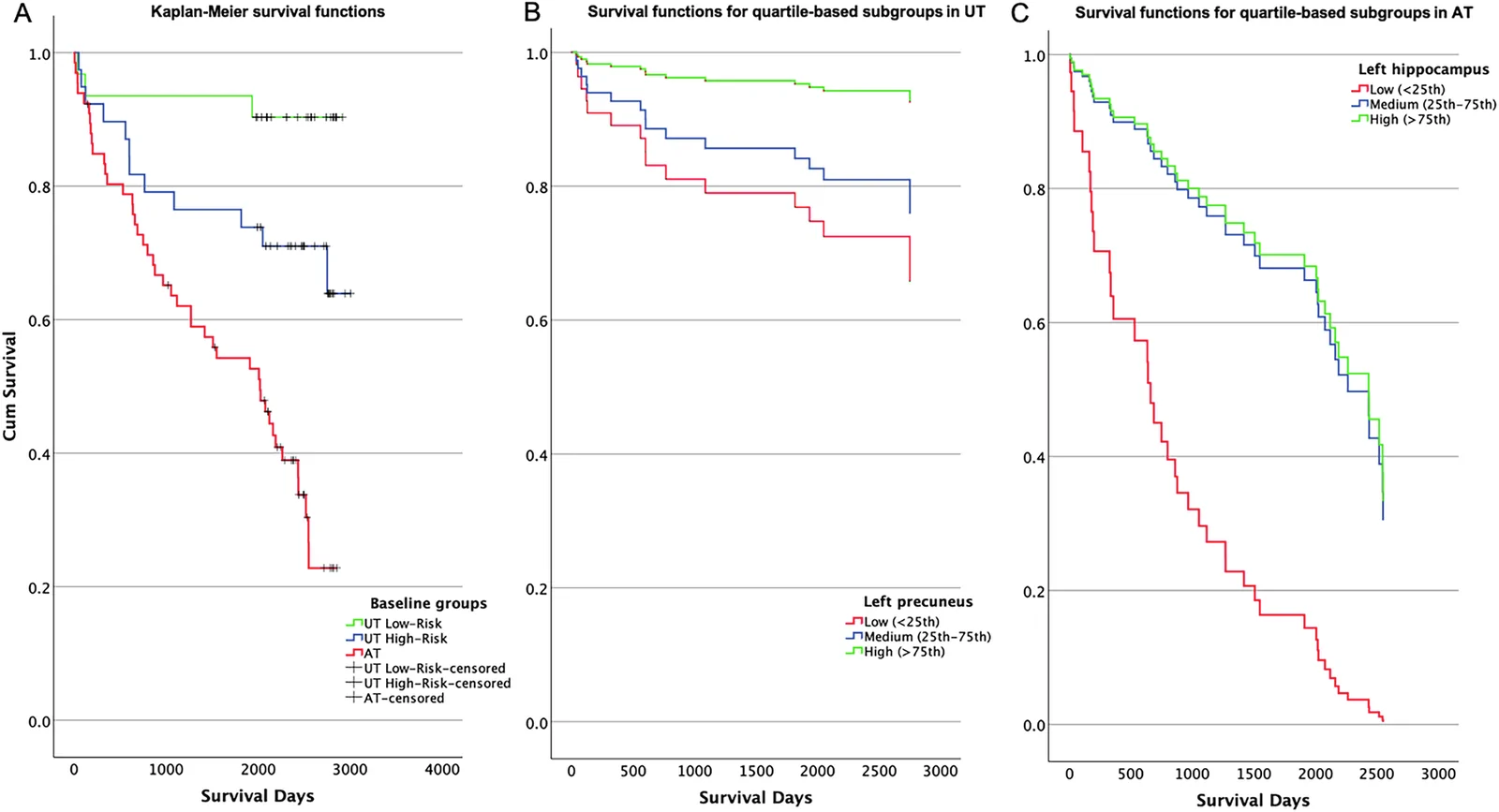
Survival functions showing the probability of illness onset or recurrence over the 7-year follow-up. (**A**) Unadjusted Kaplan–Meier curves for low-risk and high-risk unaffected twins (UT; *n* = 70) and affected twins (AT; *n* = 66) at baseline. (**B**) Adjusted survival curves for quartile-based subgroups of left precuneus volume in the UT sample. (**C**) Adjusted survival curves for quartile-based subgroups of left hippocampal volume in the AT sample.

Clinical follow-up assessments were conducted, on average, 7.1 years after baseline (SD = 0.8 years). Baseline UT (*n* = 70) and AT (*n* = 66) participants were stratified by clinical outcome (onset/recurrence during follow-up; Table 1). Among UT, 15 developed mood episodes (12 high-risk, 3 low-risk), while 44 AT experienced recurrence. Kaplan-Meier analysis revealed significantly different episode-free survival distributions across groups (low-risk UT, high-risk UT, AT; log-rank p < 0.001; Fig. 1A).

**Table 1:** Demographic and clinical comparison between subgroups with and without onset or recurrence of mood episode during the follow-up period in the affected and unaffected groups respectively.

	Unaffected Twins (*n* = 70)			Affected Twins (*n* = 66)		
	w/ onset	w/o onset	*p*	w/ recurrence	w/o recurrence	*p*
**Demographic data at baseline**	*n* = 15	*n* = 55		*n* = 44	*n* = 22	
Risk status, n (LR/UD/BD co-twin)	3/7/5	28/22/5		-	-	-
Age, yrs (SD)^a^	35.9 (9.7)	37.3 (9.8)	0.997	39.8 (8.1)	33.4 (7.8)	0.989
Sex, % women	80.0%	69.1%	0.528	75.0%	54.5%	0.104
Education, years (SD)^a^	15.8 (3.5)	15.6 (3.0)	0.888	15.0 (3.6)	14.6 (3.0)	1
Handedness, % right	87.3%	73.3%	0.233	86.4%	77.3%	0.485
Paired twins in sample, %	66.7%	70.9%	0.758	79.5%	81.8%	1
**Clinical data at baseline**						
BD-cotwin/BD, %	33.3%	9.1%	**0.023**	34.1%	59.1%	0.599
UD-cotwin/UD, %	46.7%	40.0%	**0.023**	65.9%	40.9%	0.599
Discordant time/Illness duration, yrs^a^	13.2 (8.4)	10.9 (8.3)	0.430	14.8 (8.6)	10.8 (5.1)	0.088
Taking medication, %	6.7%	1.8%	0.385	68.2%	40.9%	0.062
Months in remission (SD)^a^	-	-	-	58.9 (86.7)	47.7 (50.1)	0.594
HDRS (SD)^a^	2.1 (1.4)	2.3 (2.5)	0.735	4.5 (3.4)	4.3 (2.6)	0.723
YMRS (SD)^a^	1.7 (1.2)	1.4 (1.5)	0.630	1.9 (2.3)	1.7 (1.6)	0.619
MDI (SD)^a^	7.9 (6.5)	5.6 (4.8)	0.103	10.7 (8.3)	9.9 (8.1)	0.951
**Follow-up investigation**						
Follow-up time, days (SD)	2650 (177)	2510 (314)	**0.029**	2690 (281)	2454 (248)	**0.002**
Time to onset/recurrence, days (SD)	871 (873)	-	-	1137 (864)	-	-
**Cognitive impairment**						
SCIP, M (SEM)^a^						
VLT-I Baseline	22.5 (0.9)	23.7 (0.5)	0.281	22.8 (0.6)	22.7 (0.8)	0.567
VLT-I Follow-up	23.0 (1.2)	22.5 (0.8)		21.9 (0.7)	22.6 (1.4)	
WMT Baseline	18.6 (0.7)	19.6 (0.4)	0.433	19.4 (0.5)	19.1 (0.7)	0.549
WMT Follow-up	18.8 (1.0)	18.9 (0.6)		19.5 (0.8)	18.4 (1.3)	
VFT Baseline	16.9 (1.2)	15.3 (0.7)	**0.014**	13.9 (0.9)	14.0 (1.2)	0.512
VFT Follow-up	13.9 (1.3)	15.7 (0.8)		13.8 (0.9)	14.9 (1.5)	
VLT-D Baseline	8.0 (0.5)	7.6 (0.3)	0.767	7.3 (0.3)	7.2 (0.4)	0.136
VLT-D Follow-up	7.8 (0.6)	7.2 (0.4)		6.7 (0.4)	7.6 (0.7)	
PST Baseline	10.8 (0.6)	11.6 (0.4)	0.257	10.5 (0.5)	10.6 (0.7)	0.587
PST Follow-up	10.8 (0.5)	10.8 (0.4)		10.6 (0.4)	1.2 (0.6)	
Total Baseline	76.5 (2.8)	77.8 (1.8)	0.979	74.2 (1.9)	73.7 (2.6)	0.420
Total Follow-up	74.1 (3.1)	75.5 (2.1)		72.5 (2.3)	75.1 (4.1)	

*UD* unipolar disorder, *BD* bipolar disorder, *LR* low-risk, LQ lateral quotient, *NA* not applicable, *HDRS* hamilton depression rating scale (17-item), *YMRS* young mania rating scale, *MDI* major depression inventory, *VLT-D* verbal learning test-delayed, *VLT-I* verbal learning test-immediate, *WMT* working memory test, *VFT* verbal fluency test, *PST* processing speed test, *p* uncorrected *p* value for group effect.

^a^Statistical test accounted for within-twin pair correlation.

*p*-values in bold are significant.

UT and AT outcome subgroups were matched for demographics (age, sex, handedness, education) and depression severity (HDRS-17, YMRS, MDI). UT with BD-diagnosed co-twins had higher onset risk (50%) than those with MDD co-twins (24%) or no family history (10%) (p = 0.023). Follow-up duration was longer in onset/recurrence subgroups (UT: p = 0.029; AT: p = 0.002).

### Cox survival analyses

In the UT group, smaller left precuneus volume (HR = 1.11, p = 0.007) and thinner left (HR = 1.09, *p* = 0.023) and right (HR = 1.08, p = 0.040) SFG at baseline were significantly associated with increased hazard of mood episode onset during follow-up (Table 2). These associations remained significant when analyses were restricted to the high-risk UT subgroup (left precuneus: p = 0.003; left SFG: p = 0.043; right SFG: p = 0.05). Among AT participants, smaller left (HR = 1.11, p = 0.010) and right (HR = 1.07, p = 0.023) hippocampal volumes and right precuneus (HR = 1.05, p = 0.037) at baseline predicted higher recurrence risk (Table 2).

**Table 2:** Findings from the Cox survival analysis that included age, sex, TIV, HDRS, and brain structure predictor variables.

	Unaffected Twins (n = 70)					Affected Twins (n = 66)				
Cox predictors	*p*	*p*_corr_	HR	CI	7-year Q1/Q2-3/Q4	*p*	*p*_corr_	HR	CI	7-year Q1/Q2-3/Q4
Left Hippocampus	0.460	0.876	0.90	[0.67–1.22]	0.74/0.77/0.87	0.003	**0.010**	1.11	[1.04–1.19]	0.01/0.31/0.33
Right Hippocampus	0.719	0.986	0.90	[0.82–1.22]	0.94/0.78/0.71	0.007	**0.023**	1.07	[1.04–1.11]	0.05/0.21/0.36
Left Precuneus	0.002	**0.007**	1.11	[1.00–1.11]	0.66/0.76/0.93	0.142	0.404	1.02	[1.00–1.10]	0.05/0.25/0.04
Right Precuneus	0.056	0.177	1.11	[1.00–1.11]	0.64/0.82/0.83	0.011	**0.037**	1.05	[1.00–1.11]	0.02/0.32/0.32
Left SFG thickness	0.007	**0.023**	1.09	[1.02–1.18]	0.44/0.72/0.97	0.027	0.088	0.97	[0.95–1.00]	0.09/0.12/0.42
Right SFG thickness	0.012	**0.040**	1.08	[1.02–1.15]	0.56/0.76/0.96	0.329	0.741	0.98	[0.96–1.02]	0.26/0.27/0.12
Left ACC	0.390	0.812	1.00	[0.90–1.11]	0.81/0.73/0.92	0.091	0.276	1.00	[1.00–1.11]	0.11/0.25/0.35
Right ACC	0.773	0.993	1.00	[0.90–1.11]	0.82/0.80/0.74	0.745	0.990	1.00	[1.00–1.00]	0.11/0.21/0.20

*p*-values represent uncorrected estimates from the Cox models; *p*_*corr*_ represent the Sidak-corrected p-values (significant values in bold); HR = Hazard Ration per unit decrease (100 mm³ for volume and 0.01 mm for cortical thickness); CI = 95% confidence interval for HR. Structural predictors were grouped into quartiles, defined as low (Q1; ≤25th percentile), medium (Q2–Q3; 25th–75th percentile), and high (Q4; ≥75th percentile). The 7-year adjusted episode-free survival probabilities is presented for each quartile group.

The predicted 7-year episode-free survival probabilities for each quartile are reported for all structures in Table 2, and sample sizes for each split are provided in the Supplementary Material (Table S1). Across the structures significantly associated with increased hazard in UT, the quartile-based 7-year survival probabilities showed a broadly linear pattern, with progressively lower survival from the highest (Q4) to the lowest (Q1) quartiles (e.g., left precuneus: 0.93 → 0.76 → 0.66; left SFG: 0.97 → 0.72 → 0.44). In contrast, the AT group exhibited a clear threshold-like pattern across the hippocampal and right precuneus measures, where the lowest quartile (Q1) showed markedly reduced survival relative to the higher quartiles (e.g., left hippocampus: 0.33/0.31 vs. 0.01; right hippocampus: 0.36/0.21 vs. 0.05; right precuneus: 0.32/0.32 vs. 0.02). For brain structures that showed the most significant associations in the Cox survival analysis (left precuneus in UT and left hippocampal volume in AT), adjusted survival curves were plotted for participants in the low (Q1), medium (Q2–Q3), and high (Q4) quartiles of the respective structural measures, illustrating how lower structural values were associated with shorter episode-free survival times (Fig. 1B and C). As quartile thresholds are defined separately for each brain structure, the resulting groupings are structure-specific and survival probabilities should not be directly compared across ROIs.

The findings from the exploratory Cox survival analyses across all Desikan-Killiany atlas regions are presented in the Supplementary Material (Table S2). Several fronto-parietal regions showed associations with mood episode onset in UT and AT that may warrant further investigation, including the pre-, post-, and para-central gyri as well as the left posterior cingulate.

### Longitudinal cognitive evaluation and association with baseline brain structure

Within the UT, those who experienced onset of mood episode showed significant decline in verbal fluency (VFT; a SCIP subtest) compared with UT who remained stable (p = 0.014). No differences emerged for other SCIP subtests. In contrast, the AT remained cognitively stable from baseline to follow-up across all tests. None of the a priori selected brain ROIs tested in the Cox models were associated with the observed decline in VFT performance over time in UT (all p ≥ 0.118).

### Exploratory cortical surface analysis

**Fig. 2:**
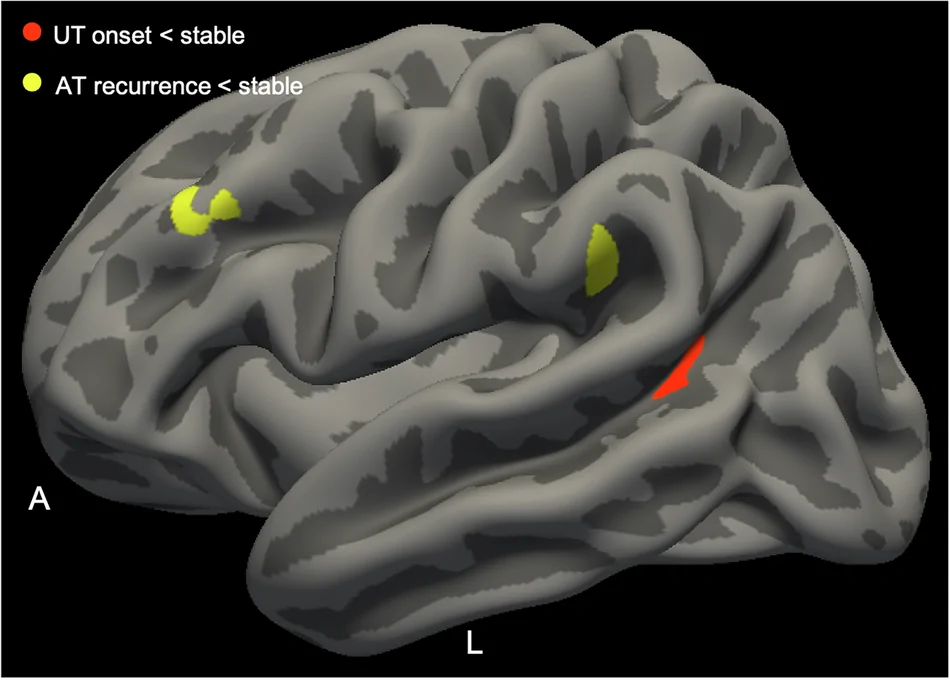
Baseline cortical structural differences associated with subsequent mood episode onset and recurrence. Regions showing lower gray matter volume and thickness at baseline in unaffected twins (UT) with onset (n = 15) compared to UT with no onset (n = 55) at follow-up (red) and in affected twins (AT) with recurrence (n = 44) compared to AT without recurrence (n = 22) at follow-up (yellow). Abbreviations: A Anterior, L left hemisphere. Cluster statistics are shown in Table 3.

Baseline vertex-wise comparisons revealed distinct neuroanatomical profiles in twins who subsequently developed mood episodes compared to those who remained stable. Among UT, participants with a future onset showed significantly smaller left superior temporal sulcus volume compared to stable UT (p = 0.011; Table 3, Fig. 2). In AT, individuals with recurrence of a mood episode exhibited reduced left supramarginal gyrus volume (p = 0.002) and thinner left rostral middle frontal cortex (p = 0.007) relative to AT who remained stable.

**Table 3:** Exploratory surface-based analysis showing significant differences in brain measures at baseline between outcome subgroups.

Contrast	Region	Measure	Size	Peak MNI coordinates			*p*
			mm^2^	x	y	z	corrected
AT recurrence <stable	Supramarginal	volume	143.82	−52.8	−34.6	26.2	0.002
AT recurrence <stable	Rostral middle frontal	thickness	127.59	−43.7	26.9	27.8	0.007
UT onset <stable	Superior temporal	volume	117.1	−45.1	−50.4	8.2	0.011

*AT* affected twins (n = 66), *UT* unaffected twins (n = 70).

## Discussion

In this large longitudinal study of 136 monozygotic twins at high risk or low risk for mood disorders (UT) and affected twins (AT), we conducted baseline clinical, cognitive, and structural MRI assessments, followed by clinical and cognitive reevaluations on average, 7.1 years after baseline. Among brain structures hypothesized to influence the risk of onset or recurrence of mood episode, thinner bilateral SFG and smaller left precuneus volume were associated with increased risk of onset (shorter episode-free survival times) in UT. Further, smaller bilateral hippocampi and right precuneus volumes were associated with increased risk of recurrence in AT. Within the UT group, individuals who experienced a mood-episode onset showed a significant decline in verbal fluency compared with those who remained stable, yet this cognitive decline was not related to baseline structural variation in the predefined ROIs. Exploratory whole-cortex analysis showed that UT with subsequent onset had reduced left superior temporal sulcus volume compared with stable UT, while AT with recurrent episodes demonstrated smaller left supramarginal gyrus volume and lower left rostral middle frontal gyrus thickness compared with stable AT.

The association between thinner bilateral SFG at baseline in UT and a higher risk of mood disorder onset followed an approximately linear gradient across structural quartiles, with progressively higher 7-year episode-free survival from Q1 (lowest thickness) to Q4 (highest thickness), which may be clinically relevant given the notable difference in 7-year survival probabilities between low- and high-thickness groups (e.g., Q1 = 0.44 vs. Q4 = 0.97 for left SFG). This suggests SFG thickness may serve as a quantifiable risk marker in high-risk populations. This association was specific to illness onset in UT and absent in recurrence among AT, possibly implying distinct neuroanatomical vulnerabilities in pre-illness stages. These results align with evidence of SFG volume reductions in first-degree relatives of mood disorder patients [12] and extend prior work by demonstrating its *prognostic* relevance. The SFG, particularly its dorsolateral and medial prefrontal cortex subdivisions, governs executive control and emotion regulation [42], and thinning in these regions may compromise emotional regulation [43] and cognitive control [44]. The specificity to UT suggests SFG integrity can buffer against initial illness onset but becomes less critical once pathological neural circuits are established. Future studies should test whether SFG-targeted interventions (e.g., cognitive training) delay onset in high-risk groups.

Interestingly, left precuneus volume was significantly associated with time to mood disorder onset in UT, whereas right precuneus volume showed significant association with time to recurrence in AT. Although this pattern may suggest hemispheric differences in precuneus function across disease stages, it should be interpreted cautiously given the moderate sample size and the strong correlation between hemispheric measures. Our findings align with prior evidence of precuneus structural differences in mood disorders. In patients with MDD, meta-analyses demonstrate precuneus volume reductions [10] and progressive thinning with depression symptoms [45]. Widespread cortical thinning, including in the precuneus, has also been reported in offspring of patients with MDD [20], suggesting these differences may represent familial risk markers rather than illness sequelae. The precuneus is a key hub of the default mode network (DMN), known to support verbal fluency, memory integration, and self-referential processing [46]. Decreased resting-state activity in the right precuneus was associated with recurrent depressive episodes in MDD [47]. This functional alteration may reflect failed compensatory suppression of the DMN, a mechanism hypothesized to mediate depression relapse risk through maladaptive rumination [48]. Interestingly, increased functional connectivity between the DMN and parts of the CEN, wer also associated with the onset of MDD [49].

Consistent with our hypothesis, smaller bilateral hippocampal volumes in AT were linked to a shorter time to recurrence. In contrast with the structures associated with first onset in UT, the association of hippocampal volume in AT was not linear across quartiles. Individuals in the lowest quartile (Q1) showed a much steeper decline in episode-free survival compared to the medium and high-volume quartiles (e.g., 7-year episode-free survival probabilities for the left hippocampus: Q1 = 0.01, Q2–Q3 = 0.031, Q4 = 0.033), suggesting that elevated recurrence risk may be driven primarily by individuals at the extreme lower end of the hippocampal volume distribution, consistent with a threshold-like rather than dose–response effect. The specificity to recurrence (rather than onset) parallels meta-analytic evidence showing hippocampal volume reductions primarily in recurrent MDD [11] suggesting cumulative stress-related neurotoxicity may underlie this association. The lack of association between hippocampal volumes and onset in UT contrasts with reports of reduced volumes in individuals at familial [18], environmental stress [50], or genetic predisposition [51]. Notably, Baaré et al. [18] found smaller hippocampal volumes in a mixed cohort of monozygotic and dizygotic high-risk UT compared to low-risk twins, with effects most pronounced in dizygotic pairs. This pattern suggests environmental factors may dominate over genetic influences in early hippocampal alterations [21]. Together, these observations imply distinct neurobiological pathways governing initial vulnerability versus illness progression. Consistent with the present findings, our prior work in this twin cohort demonstrated that the high-risk UT subgroup had enlargement of specific hippocampal subfields compared to their affected co-twins [26], reflecting either compensatory mechanisms or pre-existing protective traits which may confer resilience against illness manifestation despite shared genetic risk. This protective effect appears distinct from the volume-recurrence relationship observed in AT, which may reflect illness-related neuroplastic changes.

While smaller ACC volume has been consistently observed in MDD and BD patients [10, 13, 16], we were not able to identify any associations between ACC volume and illness onset or recurrence. The baseline structural analysis of this twin cohort [26] showed no ACC volume differences between the remitted AT and high-risk UT compared to low-risk twins. These findings suggest that ACC differences could reflect current or cumulative illness burden, including chronic stress, multiple mood episodes, or long-standing medication exposure, all of which may be less pronounced in our relatively young sample of AT, and completely absent in UT. If structural ACC differences indeed tend to emerge only after illness progression, this would make them poor predictors of future onset in individuals who have not yet experienced mood episodes. It is noteworthy that although the ACC did not emerge as a significant predictor in the primary analysis, a relatively robust association (p = 0.004) was observed between left posterior cingulate volume and recurrence risk in AT, a region functionally connected to the precuneus and a core node of the DMN [52].

Our exploratory cortical surface analysis showed that UT twins who developed mood episodes exhibited lower baseline volume in the left superior temporal sulcus, a region implicated in social-emotional processing and depression risk [53]. In AT twins, recurrence was associated with smaller left supramarginal gyrus volume and thinner rostral middle frontal cortex, findings consistent with reports of frontoparietal differences in recurrent depression [50]. The left-hemisphere predominance may reflect disruptions in networks governing language and emotional regulation, particularly cognitive strategies like reappraisal and problem-solving, a pathway increasingly recognized in mood disorder pathophysiology [54]. These findings highlight potential targets for future longitudinal research.

A key limitation is the exploratory cross-sectional cortical surface analysis, where outcome-based subgrouping (onset/recurrence) may introduce stratification bias, as it may be influenced by the baseline brain structure. While this approach does not establish causality, it helps identify pre-existing structural differences for future longitudinal research. Additionally, the absence of follow-up neuroimaging measures prevents assessing structural changes over time which could influence mood episode onset or recurrence. The general cognitive stability over time across groups suggests limited structural brain changes (neuroprogression). Another limitation is that our UT sample included both low- and high-risk twins, despite their distinct episode-free survival profiles. Pooling these groups may introduce heterogeneity, as differing baseline risk profiles could reflect distinct neurobiological mechanisms. However, separate analysis of the low-risk group was not feasible due to limited onsets. Sensitivity analysis confirmed that findings remained robust when restricting to the high-risk group, supporting the validity of the findings. Pooling also increased statistical power and captured structural differences across a broader depression risk spectrum, reflecting real-world variation. Finally, because quartile-based subgroups were defined separately for each brain measure, individual participants could fall into different categories across ROIs, which limits direct cross-structure comparisons and increases the potential for multiplicity effects. Despite these limitations, our findings underscore the potential utility of baseline neuroimaging in identifying individuals at higher risk for future mood episodes.

In conclusion, this longitudinal twin study identifies distinct neuroanatomical signatures associated with mood disorder onset and recurrence. Thinner superior frontal gyrus was linked to increased risk of onset in high-risk individuals, likely reflecting familial risk factors. In contrast, smaller hippocampal volume was associated with increased risk of recurrence in affected twins, suggesting illness-related neuroplasticity beyond shared familial risk. The stage-specific nature of these associations implies that superior frontal integrity may protect against initial illness manifestation, whereas hippocampal preservation may support sustained remission following disease onset. These findings highlight clinically actionable biomarkers for targeted interventions across different illness stages.

## Supplementary information

Supplemental Material

## Data Availability

The data that support the findings of this study are not openly available because they contain sensitive human participant clinical and imaging-derived data. Data are available from the corresponding author upon reasonable request, subject to ethical, institutional, and data protection constraints.
