# Supplementary material for: Superior frontal and hippocampal structures associated with onset versus recurrence of mood disorders in monozygotic twins
Source: Mol Psychiatry. 2026 Jun 23;31(11):6381–9. doi: 10.1038/s41380-026-03707-z (PMC13668681; doi:10.1038/s41380-026-03707-z)
Supplement: Supplementary file 1 [file 41380_2026_3707_MOESM1_ESM.docx]

**Supplementary Material for the manuscript:**

**Superior Frontal and Hippocampal Structures Associated with Onset versus Recurrence of Mood Disorders in Monozygotic Twins**

**Authors:** Julian Macoveanu, Hartwig R Siebner, Anjali Sankar, Lars Vedel Kessing, Maj Vinberg, Kamilla Miskowiak

**Image acquisition protocols**

All MRI scans were acquired at the Danish Research Centre for Magnetic Resonance (DRCMR) at Copenhagen University Hospital Hvidovre using a 3 Tesla Siemens Verio scanner (Siemens Trio, Erlangen, Germany) and a 32-channel head array receive coil. Two T1-weighted structural images were acquired using an identical 3D MPRAGE sequence (FOV =256 × 256, 224 slices, voxel size = 0.9 × 0.9 × 0.9 mm, TR = 1900 ms, TE = 2.32 ms, FA = 9°). A T2-weighted image was also acquired using a 3D spin-echo sequence (FOV = 256 × 256, 176 slices, voxel size = 1 × 1 × 1 mm, TR = 3200 ms, TE = 409 ms, FA = 120°). All scans were corrected for B0 field geometric distortions and visually inspected following image acquisition to ensure good image quality and rule out artifacts.

**Supplementary Table S1**. Supplementary details for the Cox survival analyses reported in the main text. *p*-values represent uncorrected estimates from the Cox models; *q*-values represent FDR-corrected *p*-values (significant values in bold). HR = Hazard Ration per unit decrease (100 mm³ for volume and 0.01 mm for cortical thickness). CI = 95% confidence interval for HR. Sample size indicates the number of participants in each quartile-based subgroup. Structural predictors were grouped into quartiles, defined as low (Q1; ≤25th percentile), medium (Q2–Q3; 25th–75th percentile), and high (Q4; ≥75th percentile).

|  | **Unaffected Twins (n = 70)** | | | | |  | **Affected Twins (n = 66)** | | | | |  |
| --- | --- | --- | --- | --- | --- | --- | --- | --- | --- | --- | --- | --- |
| **Cox predictors** | ***p*** | ***q*** | **HR** | **CI** | **Sample size**  **Q1/Q2-3/Q4** |  | ***p*** | ***q*** | **HR** | **CI** | **Sample size**  **Q1/Q2-3/Q4** | |
| Left Hippocampus | .460 | .613 | 0.90 | [0.67- 1.22] | 17/34/19 |  | .003 | **.024** | 1.11 | [1.04 - 1.19] | 17/35/14 | |
| Right Hippocampus | .719 | .773 | 0.90 | [0.82- 1.22] | 16/24/30 |  | .007 | **.028** | 1.07 | [1.04 - 1.11] | 18/27/21 | |
| Left  Precuneus | .002 | .**016** | 1.11 | [1.00-1.11] | 18/34/18 |  | .142 | .189 | 1.02 | [1.00 - 1.10] | 16/35/15 | |
| Right Precuneus | .056 | .112 | 1.11 | [1.00-1.11] | 15/36/19 |  | .011 | **.029** | 1.05 | [1.00 - 1.11] | 19/33/14 | |
| Left SFG thickness | .007 | **.028** | 1.09 | [1.02- 1.18] | 14/36/20 |  | .027 | .054 | 0.97 | [0.95 - 1.00] | 20/32/14 | |
| Right SFG thickness | .012 | **.032** | 1.08 | [1.02- 1.15] | 16/37/17 |  | .329 | .376 | 0.98 | [0.96 - 1.02] | 19/30/17 | |
| Left  ACC | .390 | .613 | 1.00 | [0.90- 1.11] | 15/40/15 |  | .091 | .146 | 1.00 | [1.00 - 1.11] | 19/29/18 | |
| Right  ACC | .773 | .773 | 1.00 | [0.90- 1.11] | 18/34/18 |  | .745 | .745 | 1.00 | [1.00 - 1.00] | 16/35/15 | |

**Table S2.** Findings from the exploratory COX survival analysis across the cortical regions of the Desikan-Killiany cortical atlas. *p*-values represent uncorrected estimates.

|  | **Unaffected Twins** | | | **Affected Twins** | | |
| --- | --- | --- | --- | --- | --- | --- |
| **Brain Region** | **p** | **HR** | **95% CI for HR** | **p** | **HR** | **95% CI for HR** |
| Left caudalanteriorcingulate | .966 | 1.000 | 0.819 - 1.222 | .077 | 1.105 | 1.000 - 1.222 |
| Right caudalanteriorcingulate | .526 | 1.000 | 0.905 - 1.105 | .604 | 1.000 | 0.905 - 1.105 |
| Left caudalmiddlefrontal | .520 | 1.000 | 0.905 - 1.000 | .821 | 1.000 | 1.000 - 1.000 |
| Right caudalmiddlefrontal | .827 | 1.000 | 0.905 - 1.000 | .203 | 1.000 | 0.905 - 1.000 |
| Left cuneus | .961 | 1.000 | 0.905 - 1.105 | .285 | 1.000 | 1.000 - 1.105 |
| Right cuneus | .121 | 0.905 | 0.819 - 1.000 | .700 | 1.000 | 0.905 - 1.105 |
| Left entorhinal | .222 | 1.000 | 0.905 - 1.000 | .252 | 1.105 | 0.905 - 1.350 |
| Right entorhinal | .583 | 1.000 | 0.905 - 1.105 | .085 | 1.105 | 1.000 - 1.350 |
| Left fusiform | .377 | 1.000 | 0.905 - 1.000 | .169 | 1.000 | 1.000 - 1.105 |
| Right fusiform | .331 | 1.000 | 0.905 - 1.000 | .579 | 1.000 | 1.000 - 1.105 |
| Left inferiorparietal | .041 | 1.000 | 1.000 - 1.105 | .546 | 1.000 | 1.000 - 1.000 |
| Right inferiorparietal | .234 | 1.000 | 1.000 - 1.105 | .234 | 1.000 | 1.000 - 1.000 |
| Left inferiortemporal | .912 | 1.000 | 1.000 - 1.000 | .105 | 1.000 | 1.000 - 1.105 |
| Right inferiortemporal | .312 | 1.000 | 0.905 - 1.000 | .115 | 1.000 | 1.000 - 1.000 |
| Left isthmuscingulate | .863 | 1.000 | 0.819 - 1.222 | .730 | 1.000 | 0.905 - 1.105 |
| Right isthmuscingulate | .719 | 1.000 | 0.819 - 1.222 | .245 | 1.105 | 0.905 - 1.222 |
| Left lateraloccipital | .385 | 1.000 | 0.905 - 1.000 | .343 | 1.000 | 1.000 - 1.000 |
| Right lateraloccipital | .022 | 1.000 | 0.905 - 1.000 | .321 | 1.000 | 1.000 - 1.000 |
| Left lateralorbitofrontal | .616 | 1.000 | 0.905 - 1.105 | .673 | 1.000 | 1.000 - 1.105 |
| Right lateralorbitofrontal | .872 | 1.000 | 0.905 - 1.105 | .714 | 1.000 | 1.000 - 1.105 |
| Left lingual | .638 | 1.000 | 0.905 - 1.105 | .809 | 1.000 | 1.000 - 1.000 |
| Right lingual | .549 | 1.000 | 0.905 - 1.000 | .083 | 1.000 | 0.905 - 1.000 |
| Left medialorbitofrontal | .720 | 1.000 | 0.905 - 1.105 | .582 | 1.000 | 0.905 - 1.105 |
| Right medialorbitofrontal | .415 | 1.105 | 0.905 - 1.222 | .287 | 1.000 | 1.000 - 1.105 |
| Left middletemporal | .084 | 1.000 | 1.000 - 1.105 | .241 | 1.000 | 1.000 - 1.000 |
| Right middletemporal | .055 | 1.105 | 1.000 - 1.105 | .025 | 1.000 | 1.000 - 1.105 |
| Left parahippocampal | .252 | 0.905 | 0.741 - 1.105 | .254 | 1.105 | 1.000 - 1.222 |
| Right parahippocampal | .618 | 1.000 | 0.905 - 1.222 | .786 | 1.000 | 0.819 - 1.222 |
| Left paracentral | .153 | 0.905 | 0.819 - 1.000 | .005 | 0.905 | 0.819 - 1.000 |
| Right paracentral | .114 | 0.905 | 0.819 - 1.000 | .077 | 0.905 | 0.819 - 1.000 |
| Left parsopercularis | .189 | 1.105 | 1.000 - 1.105 | .482 | 1.000 | 0.905 - 1.000 |
| Right parsopercularis | .619 | 1.000 | 0.905 - 1.105 | .635 | 1.000 | 0.905 - 1.000 |
| Left parsorbitalis | .319 | 1.105 | 0.905 - 1.350 | .545 | 1.000 | 0.819 - 1.105 |
| Right parsorbitalis | .236 | 0.905 | 0.819 - 1.105 | .935 | 1.000 | 0.905 - 1.105 |
| Left parstriangularis | .012 | 1.105 | 1.000 - 1.222 | .343 | 1.000 | 1.000 - 1.105 |
| Right parstriangularis | .363 | 0.905 | 0.819 - 1.105 | .360 | 1.000 | 1.000 - 1.105 |
| Left pericalcarine | .289 | 0.905 | 0.819 - 1.105 | .871 | 1.000 | 0.905 - 1.105 |
| Right pericalcarine | .313 | 0.905 | 0.741 - 1.105 | .995 | 1.000 | 0.905 - 1.105 |
| Left postcentral | .015 | 0.905 | 0.905 - 1.000 | .244 | 1.000 | 1.000 - 1.105 |
| Right postcentral | .796 | 1.000 | 0.905 - 1.105 | .180 | 1.000 | 1.000 - 1.105 |
| Left posteriorcingulate | .770 | 1.000 | 0.819 - 1.222 | .004 | 1.105 | 1.000 - 1.222 |
| Right posteriorcingulate | .799 | 1.000 | 0.905 - 1.222 | .822 | 1.000 | 0.905 - 1.105 |
| Left precentral | .040 | 1.000 | 0.905 - 1.000 | .717 | 1.000 | 1.000 - 1.000 |
| Right precentral | .636 | 1.000 | 0.905 - 1.105 | .031 | 1.000 | 1.000 - 1.105 |
| Left rostralanteriorcingulate | .185 | 1.105 | 1.000 - 1.222 | .656 | 1.000 | 0.905 - 1.105 |
| Right rostralanteriorcingulate | .878 | 1.000 | 0.819 - 1.105 | .999 | 1.000 | 0.905 - 1.105 |
| Left rostralmiddlefrontal | .623 | 1.000 | 0.905 - 1.000 | .855 | 1.000 | 1.000 - 1.000 |
| Right rostralmiddlefrontal | .317 | 1.000 | 0.905 - 1.000 | .849 | 1.000 | 1.000 - 1.000 |
| Left superiorfrontal | .929 | 1.000 | 1.000 - 1.000 | .555 | 1.000 | 1.000 - 1.000 |
| Right superiorfrontal | .500 | 1.000 | 1.000 - 1.000 | .279 | 1.000 | 1.000 - 1.000 |
| Left superiorparietal | .717 | 1.000 | 1.000 - 1.105 | .802 | 1.000 | 1.000 - 1.000 |
| Right superiorparietal | .188 | 1.000 | 1.000 - 1.105 | .885 | 1.000 | 1.000 - 1.000 |
| Left superiortemporal | .833 | 1.000 | 0.905 - 1.000 | .512 | 1.000 | 1.000 - 1.000 |
| Right superiortemporal | .486 | 1.000 | 1.000 - 1.105 | .322 | 1.000 | 1.000 - 1.000 |
| Left supramarginal | .120 | 1.000 | 0.905 - 1.000 | .179 | 1.000 | 1.000 - 1.000 |
| Right supramarginal | .151 | 1.105 | 1.000 - 1.105 | .171 | 1.000 | 1.000 - 1.000 |
| Left transversetemporal | .607 | 1.000 | 0.905 - 1.222 | .765 | 1.000 | 0.819 - 1.222 |
| Right transversetemporal | .112 | 1.350 | 0.905 - 1.825 | .740 | 1.000 | 0.819 - 1.222 |
| Left insula | .065 | 1.105 | 1.000 - 1.222 | .116 | 1.000 | 1.000 - 1.105 |
| Right insula | .137 | 1.105 | 1.000 - 1.105 | .517 | 1.000 | 1.000 - 1.105 |

*Note: Hazard Ratio (HR) and 95% CI are converted to reflect decrease in cortical volume using 100 mm³ units. All models adjusted for Age, Sex, Estimated Total Intracranial Volume, Hamilton Depression Rating Scores, and clinical subgroup.*
